# Post-COVID-19 Vaccination and Long COVID: Insights from Patient-Reported Data

**DOI:** 10.3390/vaccines12121427

**Published:** 2024-12-18

**Authors:** Tom C. Quach, Mitchell G. Miglis, Lu Tian, Hector Bonilla, Phillip C. Yang, Lauren Grossman, Amogha Paleru, Vincent Xin, Anushri Tiwari, Robert W. Shafer, Linda N. Geng

**Affiliations:** 1 Stanford University School of Medicine, Stanford, CA 94305, USA; tomquach@stanford.edu (T.C.Q.);; 2 Department of Neurology and Neurological Sciences, Stanford University School of Medicine, Stanford, CA 94305, USA; 3 Department of Biomedical Data Science, Stanford University, Stanford, CA 94305, USA; 4 Department of Medicine, Stanford University School of Medicine, Stanford, CA 94305, USA; 5 Case Western Reserve University School of Medicine, Cleveland, OH 44106, USA

**Keywords:** long COVID, post-acute COVID-19 syndrome, post-acute sequelae of SARS-CoV-2 infection, COVID-19 vaccination, COVID-19 immunization, COVID-19 vaccine, SARS-CoV-2 vaccination

## Abstract

Introduction: COVID-19 vaccinations reduce the severity and number of symptoms for acute SARS-CoV-2 infections and may reduce the risk of developing Long COVID, also known as post-acute sequelae of SARS-CoV-2 (PASC). Limited and heterogenous data exist on how these vaccinations received after COVID-19 infection might impact the symptoms and trajectory of PASC, once persistent symptoms have developed. Methods: We investigated the association of post-COVID-19 vaccination with any SARS-CoV-2 vaccine(s) on PASC symptoms in two independent cohorts: a retrospective chart review of self-reported data from patients (*n* = 128) with PASC seen in the Stanford PASC Clinic between May 2021 and May 2022 and a 2023 multinational survey assessment of individuals with PASC (*n* = 484). Findings: Within the PASC Clinic patient cohort (*n* = 128), 58.6% (*n* = 75) were female, and 41.4% (*n* = 53) were male; 50% (*n* = 64) were white, and 38.3% (*n* = 49) were non-white. A total of 60.2% (*n* = 77) of PASC Clinic patients reported no change in their PASC symptoms after vaccination, 17.2% (*n* = 22) reported improved symptoms, and 22.7% (*n* = 29) reported worsened symptoms. In the multinational survey cohort (*n* = 484), 380 were from the U.S., and 104 were from outside the U.S.; 88.4% (*n* = 428) were female, and 11.6% (*n* = 56) were male; and 88.8% (*n* = 430) were white, and 11.2% (*n* = 54) were non-white. The distribution of survey self-reported vaccine effects on PASC symptoms was 20.2% worsened (*n* = 98), 60.5% no effect (*n* = 293), and 19.2% improved (*n* = 93). In both cohorts, demographic features, including age, sex, and race/ethnicity, were not significantly associated with post-vaccination PASC symptom changes. There was also a non-significant difference in the median dates of COVID-19 infection among the different outcomes. BMI was significant for symptom improvement (*p* = 0.026) in the PASC Clinic cohort, while a history of booster doses was significant for symptom improvement (*p* < 0.001) in the survey cohort. Conclusions: Most individuals with PASC did not report significant changes in their overall PASC symptoms following COVID-19 vaccinations received after PASC onset. Further research is needed to better understand the relationship between COVID-19 vaccinations and PASC.

## 1. Introduction

SARS-CoV-2 vaccinations reduce the severity and number of symptoms of acute coronavirus disease 2019 (COVID-19) infections and have had an important public health impact [1,2]. COVID-19 vaccinations have been found to decrease the risk of serious adverse events, including COVID-related intensive care unit (ICU) hospitalizations, non-ICU hospitalizations, and deaths, by 63.5% and are also estimated to have prevented millions of COVID-19 deaths around the world [2,3,4]. In the U.S., COVID-19 vaccines became available in December 2020, and while guidelines and vaccination schedules have evolved over time, the current recommendation per the Centers for Disease Control and Prevention is that everyone of ages 6 months and older should receive a 2024–2025 COVID-19 vaccine, with the dosing schedule varying by age [5]. The growing literature indicates that SARS-CoV-2 vaccinations received prior to acute COVID-19 infection also reduce the risk of developing Long COVID, also known as post-acute sequelae of SARS-CoV-2 (PASC) [6]. However, there is limited and heterogenous data on how these vaccinations received after a COVID-19 infection may impact the trajectory of PASC once persistent symptoms have already developed [7].

PASC is an infection-associated chronic condition that manifests in about 5–30% of individuals infected by COVID-19 and consists of multisystemic effects lasting months and even years after initial infection [8,9]. PASC has been characterized by more than 200 symptoms, including neurological (“brain fog”, chronic fatigue syndrome, disordered sleep, etc.), cardiovascular (chest pain, palpitations, arrhythmias, etc.), and gastrointestinal (abdominal pain, diarrhea, nausea, etc.) effects, which can significantly impact people’s quality of life and function [10,11]. Studies have found that receiving two vaccine doses before the contraction of COVID-19 is associated with a decreased likelihood of persistent health consequences 12 weeks after infection when compared to no vaccination [1,10]. The findings have been heterogenous, but vaccinations have led to odds ratios of 0.22–1.03 for developing PASC [12].

While vaccination has clear public health benefits for the protection against serious acute COVID-19 illness and for the reduced likelihood of PASC, few studies have examined the impact of post-COVID-19 vaccinations on PASC symptoms. It has been suggested that vaccination may improve PASC symptoms [12], though one study on previously unvaccinated PASC patients found no significant changes in symptoms (including anosmia, respiratory distress, mental health, quality of life) in post-COVID-19 vaccinated versus unvaccinated groups at six-month follow-up evaluations [13]. Additionally, a post-vaccination syndrome has been described that mimics PASC symptoms, though its mechanism is unclear [14,15]. Understanding the potential impact of post-COVID-19 vaccination on patients with PASC has important implications not only for the clinical management of PASC and potential public health guidance but may also offer insights into the mechanisms of PASC disease pathogenesis and natural history.

In this study, we investigated the association of post-COVID-19 vaccination on PASC symptoms based on data from two independent cohorts: one from a retrospective chart review of self-reported data from patients with PASC seen in the Stanford PASC Clinic and another from a global survey study of individuals with PASC [16].

## 2. Materials and Methods

Data collected from two cohorts of individuals with PASC were used for this study.

**Figure 1:**
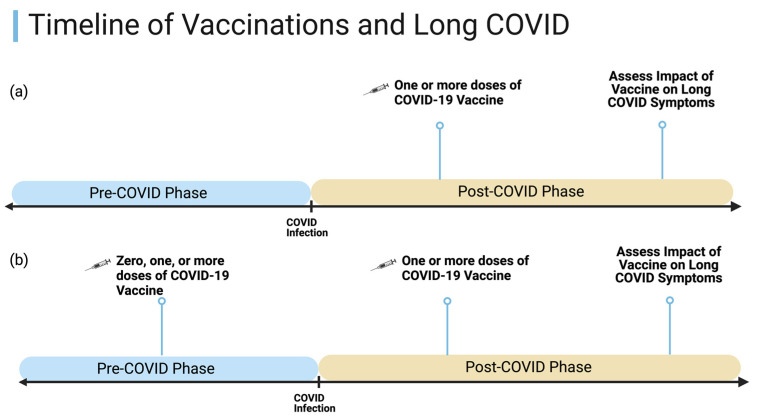
Timeline of COVID-19 vaccinations among the (**a**) Stanford PASC Clinic cohort (*n* = 128) and (**b**) multinational online survey cohort (*n* = 484). Only 2.9% of the online survey cohort (*n* = 14) received any vaccinations during the pre-COVID-19 phase.

We conducted a retrospective cohort study with electronic health record (EHR) data collected from Stanford’s PASC Clinic, known officially as the Stanford Post-Acute COVID-19 Syndrome Clinic. Clinical visits occurred during a 12-month period between May 2021 and May 2022, and a record review was conducted in September 2023 by multiple clinical research reviewers to compile demographics, PASC symptoms, COVID-19 infection and vaccination history, medical history, and patient-reported post-vaccination outcomes for symptoms. Referrals to the clinic were from both external sources and internal to Stanford, and patients were accepted from across the U.S., though most were from the state of California. The enrollment criteria for the clinic during this period included a history of SARS-CoV-2 infection, with a positive test for SARS-CoV-2 by either PCR, antigen detection, or a serology before SARS-CoV-2 vaccination, and persistent symptoms for at least 28 days following COVID-19 infection. Patients with any degree of acute infection severity were seen in the clinic, including mild outpatient cases of acute COVID-19 to severe acute critical illness. During clinical visits, patients were asked to self-report their PASC symptoms and answered a questionnaire with 29 common PASC symptoms (such as fatigue, brain fog, shortness of breath, post-exertional malaise (PEM), etc.) [17]. Patients were also asked to self-report how COVID-19 vaccinations impacted their PASC symptoms over the long-term and whether they had any acute adverse events related to the COVID-19 vaccine administration (Figure 1). Outcome variables were listed as (1) improved symptoms, (2) no change in symptoms, or (3) worsened symptoms that lasted at least 72 h or longer. Additional information such as demographics, including age, sex, and self-identified race/ethnicity, body mass index (BMI), and past medical history, including pre-existing conditions obtained at the initial visit, were also extracted from the EHR. Relevant clinical data were collected using the HIPAA-compliant database platform Research Electronic Data Capture (RedCap).

For comparison, we examined this association between post-COVID-19 vaccination and subjective impact on PASC symptoms in an independent cohort of individuals with PASC who participated in a follow-up survey from a prior international survey study assessing autonomic symptoms in PASC, as previously described [16,18]. Briefly, participants were recruited internationally through online PASC support groups and social media channels (October 2020 and August 2021), with the exclusion criteria including incomplete surveys, symptom duration < 90 days, symptom onset before November 2019, and age ≥ 65 years. Follow-up data were collected through RedCap from July to August 2023. Participants were asked to complete multiple survey instruments as previously described, including several questions that contained inquiries into the post-COVID-19 vaccine effects on PASC symptoms to assess information such as vaccination status, current symptoms, and post-COVID-19 diagnoses. These survey questions included (1) “Have you had a COVID-19 vaccine?”, (2) “Which vaccine did you received?”, (3) “If you received a vaccine not listed, which vaccine did you receive?”, (4) “Did you receive a vaccine booster(s)?”, (5) “When did you receive your last dose of the COVID-19 vaccine?”, (6) “Did your symptoms change after vaccination?”, and (7) “Were you fully vaccinated when diagnosed with the COVID infection that led to Long COVID?” [18].

### 2.1. Inclusion and Exclusion Criteria

**Figure 2:**
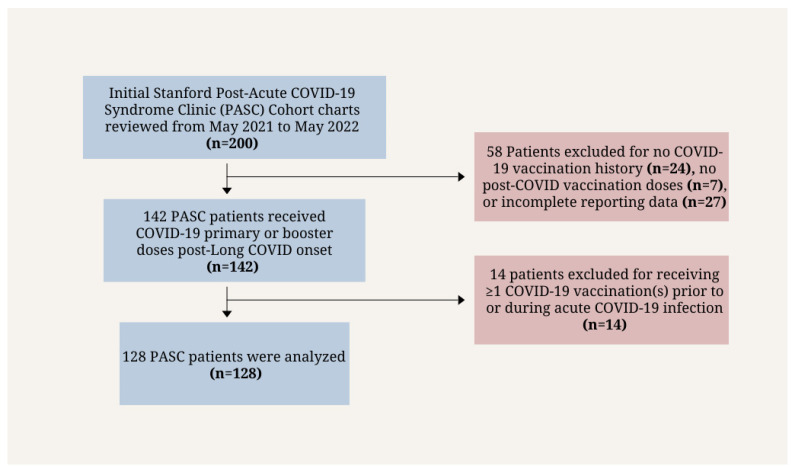
Inclusion flowchart for the Stanford PASC Clinic cohort (*n* = 128).

The analysis cohort inclusion process is shown in Figure 2. The Stanford PASC Clinic cohort’s inclusion criteria consisted of (1) current Stanford PASC Clinic patients, who had at least four weeks of persistent symptoms after COVID-19 infection, and (2) vaccination(s) after the emergence of PASC symptoms as diagnosed by Stanford PASC physicians [17]. Vaccinations from cohort individuals included mRNA (Pfizer and Moderna) and viral vector vaccines (J&J/Janssen and AstraZeneca). Vaccination effect was reported by patients during their appointment and recorded in the clinical notes by the clinician.

Regarding the independent multi-center survey cohort, participants who completed the questions asking about their vaccination history and PASC symptom trajectories were included in the final analysis for cohort comparisons [18].

### 2.2. Statistical Analyses

Among the 128 Stanford PASC patients, patient characteristics were summarized and compared across three categories according to changes in PASC symptoms after vaccination: improvement, worsening, and no change. Categorical variables (for example, sex and race) were summarized using counts and proportions and compared across the three PASC symptom change categories using a Fisher’s exact test, which evaluates contingency tables of a fixed number of rows and columns with *p*-value testing [19]. These statistical interpretation methods were also used to present findings in the multi-national survey cohort’s categorical variables. Continuous variables (for example, age and BMI) were summarized using median and quartiles and compared across the three PASC symptom change categories using an analysis of variance. Similarly, various characteristics were also summarized and compared across the three aforementioned outcome categories among the 484 survey participants. The statistical significance level was set at the two-sided 0.05 level. *p*-values reflect the significance of the statistical tests, where a *p*-value below 0.05 suggests that the observed result (proportions in the table cells) is unlikely to be due to chance, leading to the rejection of the null hypothesis of equivalence across the three outcome groups (worsened, no change, or improved) for the variables of interest (e.g., sex, race, age, BMI). In the case of a variable with multiple rows (e.g., race), then the *p*-value only reflects that this categorical variable is or is not associated with the PASC symptoms change but does not indicate which row(s) may be significantly different, and these *p* values do not indicate any particular direction or trend. R 4.1.1 (The R foundation for Statistical Computing) was used to conduct the analysis.

### 2.3. Ethics Approval

This study and corresponding protocols were approved by the Stanford Medicine Institutional Review Board (IRB).

## 3. Results

### 3.1. Stanford PASC Clinic Cohort

**Figure 3:**
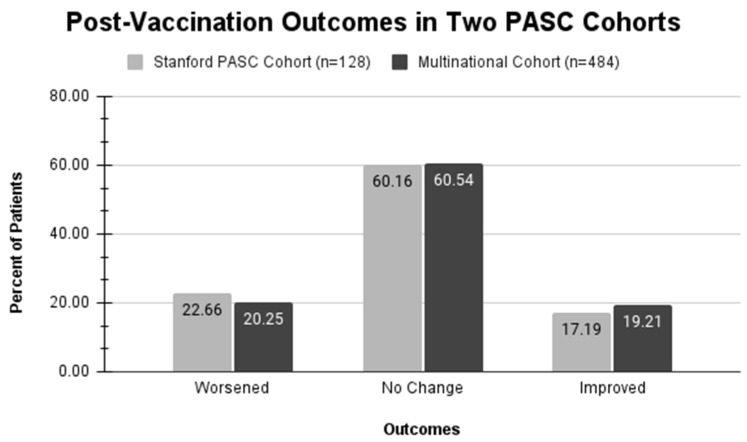
Number of individuals with PASC reporting worsened, no change, or improved PASC symptoms post-SARS-CoV2 vaccination(s) in the clinic cohort and survey cohort.

Of the 200 individuals who visited the Stanford Clinic between May 2021 and May 2022, 128 met the study’s inclusion criteria. A total of 72 were excluded for no COVID-19 vaccination history (*n* = 24), no post-COVID-19 vaccination doses (*n* = 7), receiving one or more vaccinations prior to or during their acute COVID-19 infection stage (*n* = 14), or incomplete reporting data (*n* = 27). Within the clinic patient cohort (*n* = 128), 58.6% (*n* = 75) were female, 50% (*n* = 64) white, and 38.3% (*n* = 49) non-white (Asian, Hispanic, Black, more than one race). A total of 60.2% (*n* = 77) of patients reported no change in PASC symptoms after vaccination, 17.2% (*n* = 22) reported improvement in PASC symptoms, and 22.7% (*n* = 29) reported worsening of PASC symptoms (Figure 3). The median age among patients whose symptoms had worsened, not changed, or improved was 46, 48, and 49 years, respectively.

Demographic features, including age (*p* = 0.940), sex (*p* = 0.218), and race/ethnicity (*p* = 0.557), were not significantly associated with post-vaccination PASC symptom change (Table 1). Specifically, the distribution of the proportions of patients across worsened, no change, or improved PASC symptoms did not significantly differ between males and females, suggesting that sex is not a factor associated with these three PASC symptom outcomes. Similarly, the distribution of the proportions of patients across worsened, no change, or improved PASC symptoms did not significantly differ among racial groups, suggesting that race is not a factor associated with these three PASC symptom outcomes.

Median body mass index (BMI) values were significant (*p* = 0.026) between groups, with 32.7, 28.3, and 23.4 in the improved, no change, and worsened groups, respectively. There was also a non-significant difference (*p* = 0.823) in the median dates of COVID-19 infection among the different outcomes, as well as the median days between COVID-19 infection and the first vaccine among the different outcomes (*p* = 0.738). No significant differences (*p* = 0.188) were found among patient groups receiving a varying number of vaccination doses prior to their initial PASC Clinic visit (Table 1). Specifically, the distribution of the proportion of patients across worsened, no change, or improved PASC symptoms did not significantly differ by number of vaccinations, suggesting that number of vaccinations is not a factor associated with these three PASC symptom outcomes. The clinic cohort overall had few to no pre-existing comorbid conditions, such as human immunodeficiency virus (HIV), chronic lung disease (CLD), hypertension (HTN), diabetes mellitus (DM), cardiovascular disease (CVD), and cirrhosis, and no significant differences were found among these conditions, but the subgroup numbers were low (Table 1).

In the overall clinic cohort (*n* = 128), the most common PASC symptoms reported by patients at the initial clinic visit were fatigue (89.1%), brain fog (81.3%), post-exertional malaise (80.5%), lethargy (78.1%), and unrefreshing sleep (75.8%). Among those who had reported no change in their PASC symptoms after COVID-19 vaccination (*n* = 77), the most common PASC symptoms at the initial visit were fatigue (85.7%), brain fog (79.2%), post-exertional malaise (79.2%), unrefreshing sleep (76.6%), and lethargy (75.3%). Among those who had reported improved PASC symptoms after COVID-19 vaccination (*n* = 22), the most common symptoms at the initial visit were similarly fatigue (100%), lethargy (90.9%), brain fog (86.3%), post-exertional malaise (81.8%), and unrefreshing sleep (72.7%). Among those who had worsened PASC symptoms after COVID-19 vaccination (*n* = 29), the most common symptoms at the initial visit were also fatigue (89.6%), brain fog (82.8%), post-exertional malaise (82.8%), unrefreshing sleep (75.9%), and lethargy (75.9%).

In the overall clinic cohort, 45.3% (58/128) of patients reported acute adverse events (AEs) post-vaccination typically experienced within 72 h. These included acute and often self-limiting AEs such as pain at the injection site, body aches, headaches, fatigue, fever, and lymphadenopathy, among others. Acute vaccine AEs were self-reported in 18.18% (4/22) of those who experienced improved PASC symptoms longitudinally, 37.66% (29/77) of those who experienced no change in PASC symptoms longitudinally, and 86.21% (25/29) of those who experienced worsened PASC symptoms longitudinally.

### 3.2. Multinational Survey Cohort

Of the multinational survey cohort, 484 participants who completed vaccination-related questions were included. A total of 88.4% (*n* = 428) were female, and 88.8% (*n* = 430) were white, while 78.5% (*n* = 380) of respondents were from the U.S. This group reported patterns similar to the Stanford cohort in terms of subjective PASC symptoms after post-PASC COVID-19 vaccinations. The distribution of self-reported vaccine effects on PASC symptoms was 20.2% worsened (*n* = 98), 60.5% no effect (*n* = 293), and 19.2% improved (*n* = 93) (Figure 3). The median ages among patients whose symptoms had worsened, not changed, or improved were 48.5, 50, and 48 years, respectively (Table 2).

A significant difference (*p* < 0.001) was observed across the three PASC symptom outcomes when comparing patients with booster vaccination doses. Among those who received no booster (*n* = 60), 30 (50.0%) had worsened symptoms, 25 (41.7%) had no effect, and 5 (8.33%) had improved symptoms (Table 2). In comparison, of those who received a single monovalent booster (*n* = 170), 41 (24.1%) had worsened symptoms, 107 (62.9%) had no effect, and 22 (12.9%) had improved symptoms. Among patients who received both the monovalent and bivalent booster (*n* = 254), 27 (10.6%) had worsened symptoms, 161 (63.4%) had no effect, and 66 (26.0%) had improved symptoms. There was no significant difference in the patient distribution of vaccine effects on symptoms (*p* = 0.095) across sex, racial demographics (*p* = 0.493), BMI (*p* = 0.435), and vaccination brand (*p* = 0.121). Specifically, the distribution of the proportions of patients across worsened, no change, or improved PASC symptoms did not significantly differ between males and females, suggesting that sex is not a factor associated with these three PASC symptom outcomes. Similarly, the distribution of the proportions of patients across worsened, no change, or improved PASC symptoms did not significantly differ among different racial groups, suggesting that race is not a factor associated with these three PASC symptom outcomes. The median date of COVID-19 infection across vaccination outcomes also showed no significant differences (*p* = 0.754), as well as the median number of days between COVID-19 infection and the last vaccination (*p* = 0.183). Trends were similar between the U.S. and other nations (Table 2).

## 4. Discussion

This study analyzed two independent PASC cohorts and found that most individuals with PASC did not report a subjective change in their PASC symptoms after receiving post-COVID-19 vaccination(s). The most common PASC symptoms in the Stanford PASC Clinic cohort (fatigue, brain fog, post-exertional malaise, lethargy, and unrefreshing sleep) and multinational survey cohort (fatigue, brain fog, headache, shortness of breath with exertion, and body aches) align with the existing literature regarding the commonly reported symptoms impacting individuals with PASC [10,17,18]. The nature of the data did not allow us to discern which specific symptoms might have changed after COVID-19 vaccination, if any, but we found that overall the most common PASC symptoms were similar across the patients regardless of whether they reported their symptoms not changing, improving, or worsening after vaccination. Additionally, none of the assessed sociodemographic features, clinical features including pre-existing comorbid conditions, or vaccine types were significantly and consistently associated with PASC symptom outcome. With *p*-values of greater than 0.05 when comparing subgroups, the demographics in both cohorts displayed statistical equivalence in the distribution of post-vaccination symptom outcomes. A majority of individuals in both cohorts reported no change to their PASC symptoms after their COVID-19 vaccine dose(s) post-PASC onset, regardless of age, race/ethnicity, sex, and the type of vaccine received (mRNA, viral vector, or protein subunit vaccines). Though studies examining the impact of post-COVID/post-PASC vaccination on PASC symptoms are limited, our findings are consistent with a prior study that found no significant differences in the baseline to 6-month change in anosmia, respiratory symptoms, depression, anxiety, post-traumatic stress disorder, or quality of life in patients with PASC who received a COVID-19 vaccination between baseline and 6-months and those who did not [20]. Though the overall results of our analyses did not indicate striking associations between post-COVID-19 vaccination and the variables of interest in individuals with PASC, a few subgroup findings warrant further discussion.

Although most individuals with PASC did not report changes in PASC symptoms with COVID-19 vaccination, there were small subgroups in both cohorts who reported a subjective improvement and small subgroups who reported a subjective worsening. Whether this is due to the variability inherent in subjective recall responses or underlying biological mechanisms is unclear. For those who reported subjective improvement, one could speculate hypotheses for the potential mechanisms. First, stimulation of the immune system may facilitate potential viral remnant clearance [21,22]. Persistence of viral particles is one of the leading proposed mechanisms of PASC [22], and prior data suggest that a 2-dose vaccination can reduce viral load and accelerate viral clearance in patients with SARS-CoV-2 infection and enhance the protection afforded by IgG antibodies in vivo [21]. Second, vaccination may mitigate persistent cytokine dysregulation and chronic inflammation. A prior report suggested that SARS-CoV-2 vaccination may mitigate the dysregulation of IL-1/IL-18 and gastrointestinal symptoms of PASC [23]. For those who reported worsening, one could also speculate a separate set of hypotheses. First, autoimmunity may be induced or worsened by the vaccine. Other vaccines have been known to rarely induce autoimmune conditions, and reports have also suggested this for SARS-CoV-2 vaccines [24,25,26,27,28]. Second, post-acute COVID-19 vaccination syndrome has been described in the literature with symptoms occurring after COVID-19 that overlap with PASC and other post-viral syndromes such as myalgic encephalomyelitis/chronic fatigue syndrome [29,30]. Third, nocebo effects cannot be excluded as a potential contributor to the subjective recall of worsening symptoms after vaccination. Common short-term adverse events after vaccines including COVID-19 vaccination include local site injection reactions, headache, fever, and fatigue, similar to what was reported in our study population and particularly in the patients who also reported a worsening of their PASC symptoms long-term [31,32]. The nocebo effect is well described for vaccines, and prior studies have shown that adverse event rates following vaccination were significantly higher in participants with adverse event history, lower vaccination outcome expectations, and catastrophizing tendencies related to immunization anxiety [33,34]. A systematic review and meta-analysis of COVID-19 vaccine trials found that nocebo effects accounted for over half of all reported adverse events after the first and second doses [35]. A more complete understanding of how the nocebo effect can shape study results is needed to better interpret cohort data on post-PASC vaccination outcomes.

It is also notable that a slightly larger portion of females in both cohorts reported worsening PASC symptoms following vaccination compared to males. Recent studies have found similar sex-based differences in mRNA vaccine side effects, proposing that the relationship could be accounted for by differences in the sex hormones expressed [36]. Possible mechanisms for the sex differences include a higher number of B-cells that increase antibody production in females and female sex hormones (estrogen and progesterone), stimulating larger amounts of immune cells. Hyper-reactive or lengthened reactogenicity against host antigens has been associated with more severe adverse events in the context of COVID-19 vaccinations [37]. Males, on the other hand, produce testosterone with immunosuppressive effects [38,39,40]. Females tend to have higher rates of autoimmune diseases and also PASC [41,42]. While both the cohorts in this study had higher female-to-male ratios, the clinic cohort had a relatively low reporting of pre-existing comorbid conditions, whereas 15.2% of the survey cohort participants had reported a history of an autoimmune diagnosis prior to their first SARS-CoV-2 infection [18]. This again highlights the potential complex intersection of sex-based differences, autoimmunity, PASC, and vaccination responses that warrants further research.

### Strengths and Limitations

This study’s strengths include consistent findings on post-COVID-19 vaccine effects on PASC symptoms supported across two independent cohorts, assessed using separate methodological approaches. For the survey cohort, data were collected from international participants representing geographic diversity. For the clinic cohort, patients were evaluated at a specialized PASC center and assessed clinically by a healthcare provider to have PASC.

This study had several limitations. For the clinic cohort, data were collected from a single academic medical center site with a limited sample size and sociodemographics that generally reflect the region. Referrals and the clinic population may be biased toward those who have fewer barriers to care and adequate insurance coverage. The multinational survey contained respondents who may have received a COVID-19 vaccination prior to their acute infection, leading to a non-standardized population (compared to the Stanford-analyzed cohort which included only individuals who received vaccination doses post-acute infection). Conclusions regarding the causation of the effects of vaccination on patients cannot be drawn, and generalizability may be limited. Individual symptoms could not be longitudinally tracked over time to discern how vaccination specifically affects the trajectory. While our study did not directly assess autoimmune disorders arising from PASC or COVID-19 vaccination, patients in the study reported symptoms that overlap with inflammatory conditions such as myalgic encephalomyelitis/chronic fatigue syndrome, fibromyalgia, and postural orthostatic tachycardia syndrome, which are recognized as subtypes of PASC [10,17,18]. Furthermore, those within the study cohort had immunization histories with varying numbers of vaccination doses between the onset of PASC and time of self-reporting. Thus, the dose number examined for specific post-vaccination effects was inconsistent among cohort individuals, which confounds the vaccine dose number correlation with the observed outcome for a given patient. Additional limitations include patient recall bias and subjective differences in estimating the magnitude of improvement or worsening of PASC symptoms due to the nature of the retrospective studies and lack of validated biomarkers for PASC. No data were present regarding the timing between symptom changes and immunization. Furthermore, beneficial improvements post-vaccination could be attributed to natural recovery trajectories due to the longitudinal time frames studied, and negative effects could be related to short-term side effects of the vaccine that were misinterpreted as long-term effects [43]. Similarly, reduced symptoms among those receiving more vaccinations were likely confounded by the fact that those with more severe symptoms post-immunization were less likely to receive additional vaccinations. Furthermore, we cannot account for the potential confounding effects of concomitant vaccinations that individuals may have received alongside their COVID-19 immunizations, such as the influenza vaccination.

Follow-up studies, including those that are prospective and with larger and more diverse populations, may help add context regarding the effects of differing demographics, as well as help strengthen the findings from these cohorts. Specifically, there have been hypothesized sex-based differences in Long COVID, warranting further investigation for potential differences in vaccine-related effects [44].

## 5. Conclusions

A majority of individuals with PASC in the two cohorts analyzed did not report significant changes in overall PASC symptoms following COVID-19 vaccinations given after the onset of PASC. When used as preventive measures, COVID-19 vaccinations offer protection against severe acute COVID-19 infection and are associated with a reduced risk of developing PASC. Further research is needed to determine the impact of COVID-19 vaccinations on PASC symptoms.

## Figures and Tables

**Table 1:** Association of demographics and SARS-CoV-2 mRNA vaccine timing with PASC symptoms in 128 Patients with Long COVID at the Stanford PASC Clinic.

	Worse (*n* = 29)	Same (*n* = 77)	Improved (*n* = 22)	*p*-Value *
** Proportion of Patients **	22.7%	60.2%	17.2%	
**Age** **Median (Quartiles)**	46 (32, 56)	48 (35, 59)	49 (43.3, 56.3)	0.940
** Sex **				0.218
M (*n* = 53)	8 (27.6%)	36 (46.8%)	9 (40.9%)	
F (*n* = 75)	21 (72.4%)	41 (53.2%)	13 (59.1%)	
** Race **				0.557
White (*n* = 64)	16 (55.2%)	36 (46.8%)	12 (54.5%)	
Asian (*n* = 22)	5 (17.2%)	11 (14.3%)	6 (27.3%)	
Hispanic (*n* = 18)	2 (6.9%)	14 (18.2%)	2 (9.1%)	
Black (*n* = 8)	2 (6.9%)	6 (7.8%)	0 (0.0%)	
More than one race (*n* = 1)	1 (3.4%)	0 (0.0%)	0 (0.0%)	
Unknown (*n* = 15)	3 (10.3%)	10 (13.0%)	2 (9.1%)	
**BMI** **Median (Quartiles)**	23.4 (21.5, 30.9)	28.3 (22.9, 33.0)	32.7 (27.4, 36.1)	0.026
** Comorbidities **				
HIV	0 (0%)	0 (0%)	0 (0%)	
CLD	3 (10.3%)	13 (16.9%)	2 (9.1%)	0.629
HTN	5 (17.2%)	19 (24.7%)	4 (18.2%)	0.757
DM	2 (6.9%)	5 (6.5%)	1 (4.5%)	1.000
CVD	2 (6.9%)	2 (2.6%)	0 (0%)	0.349
Cirrhosis	1 (3.4%)	0 (0%)	0 (0%)	0.398
** Median COVID date **	7 December 2020	20 December 2020	21 December 2020	0.823
** Days between COVID and 1st vaccine** **Median (Quartiles) **	115 (92, 168)	122 (80, 192)	120 (92, 192)	0.738
**Number of vaccinations**				0.188
1 (*n* = 14)	7 (24.1%)	6 (7.8%)	1 (4.5%)	
2 (*n* = 80)	17 (58.6%)	48 (62.3%)	15 (68.2%)	
3 (*n* = 32)	5 (17.2%)	22 (28.6%)	5 (22.7%)	
4 (*n* = 2)	0 (0.0%)	1 (1.3%)	1 (4.5%)	

* The statistical significance is set at the two-sided 0.05 level.

**Table 2:** Association of demographics and SARS-CoV-2 vaccine timing with PASC symptoms in 484 participants in a multinational survey cohort.

	Worse (*n* = 98)	Same (*n* = 293)	Improved (*n* = 93)	*p*-Value *
**Proportion of Patients**	20.2%	60.5%	19.2%	
**Age Median** **(Quartiles)**	48.5 (41.3, 57.0)	50.0 (41.3, 56.0)]	48.0 (2.0, 55.0)	0.823
**Sex**				0.095
Male (*n* = 56)	10 (10.2%)	29 (9.9%)	17 (18.3%)	
Female (*n* = 428)	88 (89.8%)	264 (90.1%)	76 (81.7%)	
**Race**				0.493
White (*n* = 430)	86 (87.8%)	264 (90.1%)	80 (86.0%)	
Other (*n* = 54)	12 (12.2%)	29 (9.9%)	13 (14.0%)	
**Country**				0.308
USA (*n* = 380)	72 (73.5%)	236 (80.5%)	72 (77.4%)	
Other (*n* = 104)	26 (26.5%)	57 (19.5%)	21 (22.6%)	
**BMI Median (Quartiles)**	25.2 (22.0, 29.7)	25.7 (23.2, 30.5)	26.6 (23.2, 30.5)	0.435
**Median COVID date**	11 July 2020	17 July 2020	29 May 2020	0.754
**Vaccine type**				0.121
Pfizer (*n* = 268)	52 (53.1%)	159 (54.3%)	57 (61.2%)	
Moderna (*n* = 162)	32 (32.7%)	98 (33.4%)	32 (34.4%)	
J&J (*n* = 27)	8 (8.2%)	19 (6.5%)	0 (0.0%)	
AstraZeneca (*n* = 24)	6 (6.1%)	15 (5.1%)	3 (3.2%)	
Other (*n* = 3)	0 (0.0%)	2 (0.7%)	1 (1.1%)	
**Days between COVID and last vaccine** **Median (Quartiles)**	412.5 (207, 621.8)	682 (423, 858.8)	687.5 (527.5, 919.3)	0.183
**Boosters**				<0.001
No	30 (30.6%)	25 (8.5%)	5 (5.4%)	
Single monovalent booster	41 (41.8%)	107 (36.5%)	22 (23.7%)	
Both monovalent and bivalent booster	27 (27.6%)	161 (54.9%)	66 (71.0%)	

* The statistical significance is set at the two-sided 0.05 level.

## Data Availability

The data presented in this study are available on request from the corresponding author due to the data consisting of sensitive personal health information.
